# Cellular Schwannoma of the Palate Simulating as Malignant Peripheral Nerve Sheath Tumor: A Diagnostic Marathon

**DOI:** 10.30476/dentjods.2024.101035.2265

**Published:** 2024-12-01

**Authors:** Manas Bajpai, Amit Mani, Saurabh L Sabnis, Vatchala Rani RM

**Affiliations:** 1 Dept. of Oral and Maxillofacial Pathology and Oral Microbiology, Rural Dental College Pravara Institute of Medical Sciences, Loni (Maharashtra), India; 2 Dept. of Periodontology, Rural Dental College, Pravara Institute of Medical Sciences, Loni (Maharashtra) India; 3 Dept. of Oral and Maxillofacial Pathology and Oral Microbiology, Faculty of Dentistry, Jamia Millia Islamia, New Delhi, India

**Keywords:** Schwannoma, Malignant peripheral nerve sheath tumor, Oral neoplasms, Neurilemmoma, Immunohistochemistry

## Abstract

Schwannomas are considered benign soft tissue tumors that originate from Schwann cells. Oral Schwannomas are rare and account for only 1% of all Schwannomas. Cellularschwannoma (CS) is a rare histological variant of schwannoma, characterized by high cellularity and cellular atypia. We present a case of localized growth of palatal mucosa that imitated the features of malignant peripheral nerve sheath tumor (MPNST) on histopathological examination; it was differentiated from MPNST by the correlation of clinical, histopathological, and immunohistochemical features.

## Introduction

Oral schwannomas (Neurilemmomas) are rare benign painless soft tissue neoplasms that account for only 1% of all Schwannomas [ 1 ]. Intra-orally, these lesions tend to mimic the clinical features of other soft tissue tumors such as lipoma, peripheral giant cell granuloma, peripheral ossifying fibroma, and fibroma. Histopathologically, they are divided into cellular schwannoma (CS) and ancient schwannoma [ 2 ]. CS is characterized by hypercellular areas made up of a collection of numerous spindle-shaped cells arranged in fascicles, with a predominant Antonitype A pattern along with minimal verocay bodies [ 3 ]. CS may also show nuclear atypia, hyperchromasia, and focal areas of necrosis, large sinusoidal elongated blood vessels, and a herringbone pattern of spindle-shaped cells. The diagnosis of CS solely on histopathology is challenging due to the features similar to malignant peripheral nerve sheath tumor (MPNST), solitary fibrous tumor (hemangiopericyoma), fibrosarcoma, and myofibrosarcoma [ 4 ].

We report a rare case of CS of the palate, diagnosed by meticulous immunohistochemical analysis. The present report describes the importance of immunohistochemistry in diagnosing CS and discusses various histopathological differential diagnoses of CS.

## Case Presentation

**Figure 1:**
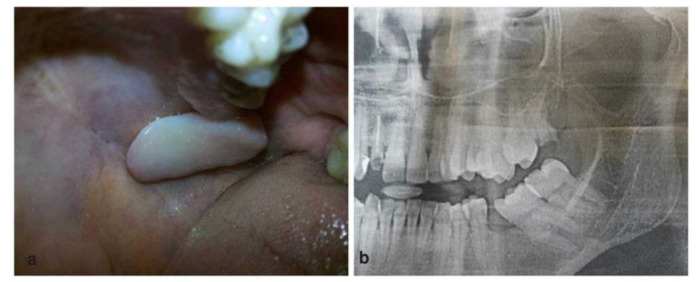
**a:** Clinical picture of the lesion presented on the posterior palate, **b:** Panoramic radiograph reveals that lesion was not associated with the underlying bone

An otherwise healthy 33-year-old lady presented to our Rural Dental College Loni (India) in February 2023 for the evaluation of a localized growth in her upper, left-back region of the jaw for 1 year. The family history and personal history of the patient were non-contributory to the presenting symptom. Intra-oral examination revealed localized soft tissue growth measuring about 3×2cm (CM) extending from the maxillary tuberosity to the midline of the hard palate. The color of the growth was pink, similar to that of the adjacent mucosa, without showing signs of inflammation or discharge (Figure 1a). On palpation, the swelling was found to be soft and fluctuant and it was not indurated. 

A panoramic radiograph revealed that the growth was not attached to the underlying bone; no resorption of the bone was noted (Figure 1b).

The lesion was provisionally diagnosed as fibroma with the differential of a benign salivary gland tumor. The incisional biopsy was performed, and the excised tissue was sent for histopathological evaluation.

**Figure 2:**
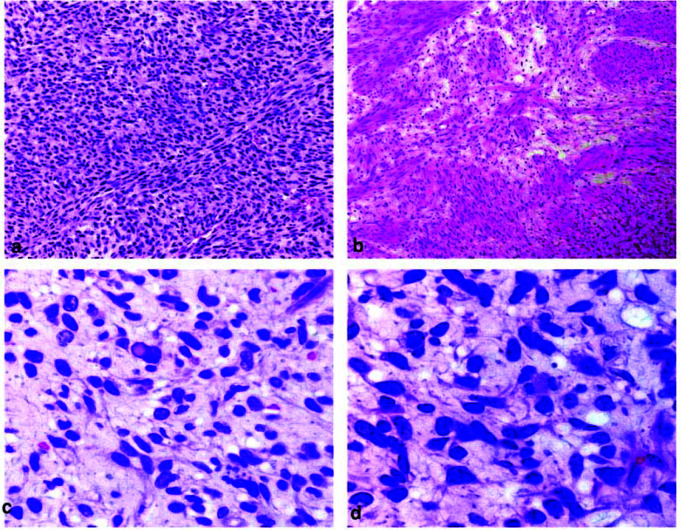
**a:** Hypercellular area made up collection of numerous spindle shaped cells arranged in fascicles (Hematoxylin and eosin staining 20×), **b:** Spindle shaped cells show palisading arrangement resembling Antoni A type of arrangement (Hematoxylin and eosin staining 40×), **c:** Degenerative cytological atypia consisted of irregular, multilobed, and hyperchromatic nuclei with intra -nuclear vacuoles. (Hematoxylin and eosin staining 40×), **d:** Spindle shaped cells show variable mitotic activity but without atypical mitotic figure (Hematoxylin and eosin staining 100×)

The histopathological examination of the hematoxylin and eosin-stained soft tissue section revealed a hypercellular area made up of sheets of spindle-shaped cells arranged in fascicles (Figure 2a) with Antonitype A arrangement (Figure 2b); degenerative cytological atypia was noted comprised of irregular, multilobed, hyperchromatic nuclei with intranuclear vacuoles (Figure 2c), spindle-shaped cells with a wavy nucleus, nuclear pleomorphism, nuclear hyperchromatism, and variable mitotic figures (Figure 2d).

The diagnosis of aggressive neural tumors was given on histopathological evaluation with the suspicion of malignant peripheral nerve sheath tumor (MPNST), fibrosarcoma, myofibrosarcoma, and solitary fibrous tumors. In order to reach the final diagnosis, immunohistochemical staining was performed using S 100, smooth muscle actin (SMA), Desmin, Ki 67, and p53.

**Figure 3:**
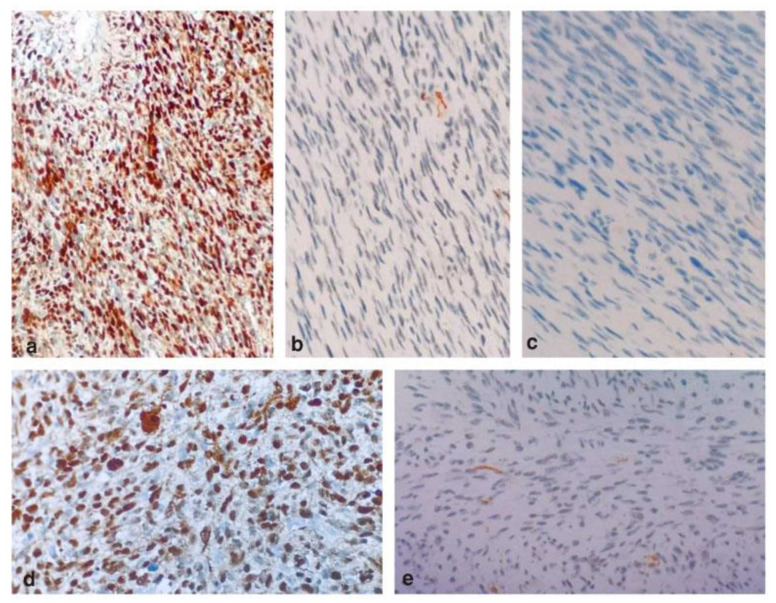
Immunohistochemical images showing tumor cells expression for various markers, **a:** Positive expression of the tumor cells for S -100, **b:** Negative expression of the tumor cells for Actin, **c:** Negative expression of the tumor cells for Desmin, **d:** The tumor cells showed positive expression for Ki 67 with more than 50% positive cells, **e:** The tumor cells showed negative expression for p53

The tumor showed a strong positive expression for the S-100 marker (Figure 3a), thus confirming its neural origin. The tumor showed negative expression for SMA and Desmin (Figures 3b and 3c), ruling out the possibility of myofibrosarcoma. The tumor showed positive expression for Ki67 with more than 50 percent positivity (Figure 3d), depicting its aggressive nature. The lesion showed negative expression for p53, excluding the probability of malignancy (Figure 3e).

The final diagnosis of CS was made. The treatment plan consisted of a complete surgical excision of the lesion completely. A wide surgical excision of the lesion was performed, including the 5mm margins around the lesion, under local anesthesia. The gross tissue was pink in color with soft consistency.

After a complete excision, suturing of the wound was performed using 4.0 vicryl suture material. The follow-up period of 8 months (the patient is still on follow-up) was uneventful. A written consent from the patient was obtained for the publication of this report.

## Discussion

Schwannoma is a slow-growing, benign tumor of neural origin. Histopathologically, it is classified into seven different variants, including classical, plexiform, granular, melanotic, ancient schwannoma, and CS [ 3 - 5 ]. The present case revealed a histopathological and immunohistochemical profile consistent with CS. Schwannomas commonly occur in the head and neck regions; however, their occurrence in the oral cavity is exceedingly rare, accounting for only 1% of all schwannomas. Intra-orally, the tongue is the commonest site. An exhaustive literature review revealed only five cases of intraoral CS [ 6 ] (Table 1). This is the sixth case of intraoral CS, to the best of our knowledge, and only the second case affecting the palate. There is a paucity of information about intra-oral CS pertaining to race, age, and gender predilection. In the present case, the patient was a 33-year-old female. The etiology of intraoral CS is not known; it is believed that they develop from Schwann cells. Intraoral CS is usually asymptomatic, and out of five previously reported cases, only one presented as painful growth [ 7 ]. Intra-oral CS can mimic various slow-growing benign tumors of the oral cavity, such as lipoma, fibroma, peripheral ossifying fibroma, and so on [ 4 ]. Histopathologically, the differential diagnoses of CS are classical schwannoma, ancient schwannoma, neurothakeoma, and MPNST (Table 2).

**Table 1:** Review of the cases of cellular schwannoma reported in the oral cavity

Serial No	Reference	Age/gender	Location	Clinical Findings	Immunohistochemistry
1	Redman RS *et al*. 1996 [ 14 ]	31/F	Mandibular canal	Asymptomatic	S- 100 positivity
2	Koizumi Y *et al*.2002 [ 2 ]	34/M	Buccal mucosa	Asymptomatic	S- 100 and vimentin positivity
3	Bhalerao S *et al* 2012 [ 4 ]	38/F	Palate, maxillary gingival and mandibular gingival	Asymptomatic	Vimentin positivity
4	Poonja PA *et al*. 2017 [ 6 ]	30/F	Hard palate	Asymptomatic	S- 100 positivity
5	Pandian A *et al*. 2022 [ 3 ]	44/F	Buccal mucosa	Asymptomatic	S-100 positivity
6	Bagul S *et al*.2023 [ 7 ]	27/M	Gingiva	Asymptomatic	S-100, Ki 67 and vimentin positivity
					p 53 negativity

**Table 2:** The differential diagnoses of cellular schwannoma

S.NO	Differential diagnosis	Differentiating features
1	Classic schwannoma	Classic schwannomas show both AntoniA and B patterns with verocay bodies. Cellular schwannomas generally shows predominantly AntoniA area without verocay bodies, nuclear hyperchromatism, and higher mitotic activity
2	Ancient schwannoma	Ancient schwannomas show degenerative features i.e., haemorrhagic areas and thrombus formation
3	Neurothakeoma	Neurothakeomas generally show areas of myxoid degeneration
4	Malignant peripheral nerve sheath tumor	MPNST generally shows perivascular hypercellularity, vascular invasion, and areas of necrosis with abundant abnormal mitotic figures and Immunohistochemically they show strong positive expression for P53 marker

The present case showed Antoni type A cell arrangement along with nuclear pleomorphism and nuclear hyperchromasia with increased mitotic activity. The present case also showed large dilated blood vessels with an area of myxoid degeneration. The diagnosis of intraoral CS solely on a histopathological basis is difficult due to the spindle cell fascicles, nuclear pleomorphism, nuclear hyperchromatism, and increased mitotic activity [ 3 , 8 ]. It imitates malignant tumors, i.e., MPNST, fibrosarcoma, myofibrosarcoma, and synovial sarcoma, and for that very reason, Fletcher CD *et al*. [ 9 ] described CS as a pseudosarcomatous entity. Unlike these malignant tumors, CS is known to show positive expression for S-100 and an unevenly distributed reactivity for glial fibrilar acidic protein [ 5 , 10 ]. Ki67 is a commonly used immunohistochemical marker to predict proliferation rate of tumors including the nerve sheath-derived neoplasms and it is helpful to differentiate them from their malignant counterparts [ 11 ]. Pakemzci M *et al*. [ 12 ] compared the expression of Ki 67 between MPNST and CS; they found that MPNST had higher Ki 67 proliferation indices.

In the present case, a meticulous immunohistochemical analysis was also performed using S-100, Ki-67, SMA, Desmin, and P53. The tumor showed a positive reaction for S-100 and Ki-67, revealing its neural origin and high mitotic activity, a negative reaction for SMA and Desmin excluding the diagnosis of myofibrosarcoma; and a negative expression for p-53, ruling out the possibility of malignancy [ 13 ].

Local surgical excision is the most preferred treatment for CS; recurrence and malignant transformation have not yet been reported [ 5 - 6 , 14 ].

## Conclusion

CS is a pseudosarcomatous tumor, which tends to mimic various aggressive tumors on histopathology. Immunohistochemistry is required to reach the final diagnosis and to exclude the risk of aggressive treatment modalities associated with malignant tumors.
